# Predictors of Mortality and Prehospital Monitoring Limitations in Blunt Trauma Patients

**DOI:** 10.1155/2015/983409

**Published:** 2015-02-01

**Authors:** Matej Strnad, Vesna Borovnik Lesjak, Vitka Vujanović, Tine Pelcl, Miljenko Križmarić

**Affiliations:** ^1^Prehospital Unit, Center for Emergency Medicine, Community Health Center Maribor, Ulica Talcev 9, SI-2000 Maribor, Slovenia; ^2^Medical Faculty, University of Maribor, Taborska Ulica 8, SI-2000 Maribor, Slovenia; ^3^Faculty of Health Sciences, University of Maribor, Žitna Ulica 15, SI-2000 Maribor, Slovenia

## Abstract

This study aimed at determining predictors of in-hospital mortality and prehospital monitoring limitations in severely injured intubated blunt trauma patients. We retrospectively reviewed patients' charts. Prehospital vital signs, Injury Severity Score (ISS), initial Glasgow Coma Scale (GCS), Revised Trauma Score (RTS), arterial blood gases, and lactate were compared in two study groups: survivors (*n* = 40) and nonsurvivors (*n* = 30). There were no significant differences in prehospital vital signs between compared groups. Nonsurvivors were older (*P* = 0.006), with lower initial GCS (*P* < 0.001) and higher ISS (*P* < 0.001), along with higher lactate (*P* < 0.001) and larger base deficit (BD; *P* = 0.006), whereas RTS (*P* = 0.001) was lower in nonsurvivors. For predicting mortality, area under the curve (AUC) was calculated: for lactate 0.82 (*P* < 0.001), for ISS 0.82 (*P* < 0.001), and for BD 0.69 (*P* = 0.006). Lactate level of 3.4 mmol/L or more was 82% sensitive and 75% specific for predicting in-hospital death. In a multivariate logistic regression model, ISS (*P* = 0.037), GCS (*P* = 0.033), and age (*P* = 0.002) were found to be independent predictors of in-hospital mortality. The AUC for regression model was 0.93 (*P* < 0.001). Increased levels of lactate and BD on admission indicate more severe occult hypoperfusion in nonsurvivors whereas vital signs did not differ between the groups.

## 1. Introduction

Traditional vital signs could be relatively insensitive to hypovolemia [1] and patients with already hypoperfused tissue could appear to be haemodynamically stable with normal vital signs [2]. Tissue hypoperfusion due to different medical conditions or trauma may lead to multiorgan failure and can worsen prognosis in these patients. Severely injured trauma patients are at significant risk of tissue hypoperfusion due to hypovolemic shock. Many studies have evaluated lactate levels as a surrogate of tissue hypoperfusion to predict outcome in severely injured patients [3–6]. Raised lactate levels as a product of anaerobic metabolism are found when mismatch between oxygen supply and demand occurs and are associated with increased mortality in trauma patients [2–5]. Lactate [7] and base deficit [8] monitoring are useful markers to guide trauma patient's resuscitation and are also used to assess adequacy of resuscitation from prehospital setting [9] to intensive care unit (ICU) [10].

Different types of study populations were studied to assess the ability of lactate levels to predict outcome. It was evaluated in ICU with both trauma and medical patients included [11], in ICU with only medical patients included [10], in prehospital setting in patients with medical complaints [12], in trauma population including both penetrating and blunt injuries [6, 13], in elderly normotensive blunt trauma patients [5], and also in pediatric population [14]. The purpose of this single center retrospective study was to determine (a) the predictors of in-hospital mortality and (b) prehospital monitoring limitations in severely injured adults with multiple injuries who sustained blunt trauma and were intubated in prehospital setting.

## 2. Materials and Methods

### 2.1. Statement of Human Rights

The study was approved by the National Medical Ethics Committee of the Republic of Slovenia, deciding the study can be conducted without informed consent from participants.

### 2.2. Study Design

This retrospective, observational study was conducted in Maribor, Slovenia, and adjacent rural areas encompassing a population of about 200.000 inhabitants. The Center for Emergency Medicine in Maribor hosts the Maribor Emergency Medical Service (EMS) system, which also includes two emergency physician-led teams. We retrospectively reviewed medical charts of 70 consecutive blunt trauma patients with multiple injuries who underwent prehospital endotracheal intubation (ETI) using rapid sequence intubation (RSI) method between January 2000 and December 2012 and were 18 years of age or older. Patients with isolated severe head injury or penetrating injuries were not included. Patients were endotracheally intubated for several reasons: initial Glasgow Coma Scale (GCS) less than 8, compromised airway, ensuring adequate ventilation, and so forth. Endotracheal placement of the tube was confirmed by measuring partial pressure of end tidal carbon dioxide (petCO_2_)* in vivo*. Patients were mechanically ventilated after ETI. Parameters (minute volume, respiratory frequency) on transport ventilators were selected by emergency physicians. Trauma patients were treated according to the current trauma life support guidelines and were directly transported to the Emergency Department (ED) and then admitted to the surgical ICU of the University Clinical Center of Maribor. The study sample was subdivided into two groups:* survivors* and* nonsurvivors* at hospital discharge. Prehospital vital signs (petCO_2_, heart rate, mean arterial pressure, respiratory rate, and arterial oxygen saturation) and initial GCS were measured at the scene. All laboratory testing and arterial blood gases analysis were performed at the arrival in the ED in the central hospital laboratory. Intervention time was defined as a time interval from the time of call until arrival in the ED. Injury severity was assessed by the Injury Severity Score (ISS) and Revised Trauma Score (RTS).

### 2.3. Statistical Analysis

Statistical analysis was undertaken with IBM SPSS software, version 22 for Windows (SPSS, Chicago, Illinois). Normality was evaluated with the Kolmogorov-Smirnov and the Shapiro-Wilk test. The continuous data are presented as median with interquartile range (IQR) and were compared between groups using the Mann-Whitney* U* test. Categorical variables are presented as counts (percentage). Differences between categorical variables were analysed by Fisher exact test. To evaluate the prognostic accuracy for in-hospital mortality of lactate levels, ISS and BD receiver operating characteristic (ROC) curves were constructed. For each variable, area under the curve (AUC) of sensitivity plotted against 1−specificity is reported with 95% confidence intervals (95% CI). ROC curve analysis was used for each of three variables to determine the optimal cut-off point that maximized desired test properties. The optimal cut-off of the investigated parameters was calculated as the threshold value with the highest specificity and sensitivity. For the cut-off points, results are presented as sensitivity, specificity, and positive and negative predictive values (PPV and NPV, resp.).

Selected variables identified during univariate analysis were then subjected to multivariate analysis logistic regression model to determine independent predictors of mortality. We developed a multivariate logistic regression model to ascertain the effects of lactate, ISS, BE, pCO_2_, age, and initial GCS on the likelihood that participants will not survive. Initial GCS instead of RTS was used in regression model. Since there were statistically significant differences in initial GCS between compared groups and not in blood pressure and respiratory rate, we can assume that initial GCS mostly contributed to the significant difference in RTS. Odds ratios (OR) and their 95% confidence interval (CI) were calculated. The strength of the relationship between lactate and base deficit was analysed with Pearson's correlation coefficient (*r*) and coefficient of determination (*R* ^2^). A clinically relevant association was defined as *R* ^2^ > 0.5. All *P* values were two sided and a value of *P* < 0.05 was considered statistically significant.

## 3. Results

Patients' characteristics along with prehospital vital signs, medications, and intravenous fluids used for prehospital resuscitation are presented in Table 1. Patients sustained blunt trauma mostly due to motor vehicle collisions and falls. There were statistically significant differences between survivors and nonsurvivors in age, lactate, RTS, ISS, BD, initial GCS, and arterial pressure of carbon dioxide (pCO_2_).

**Figure 1:**
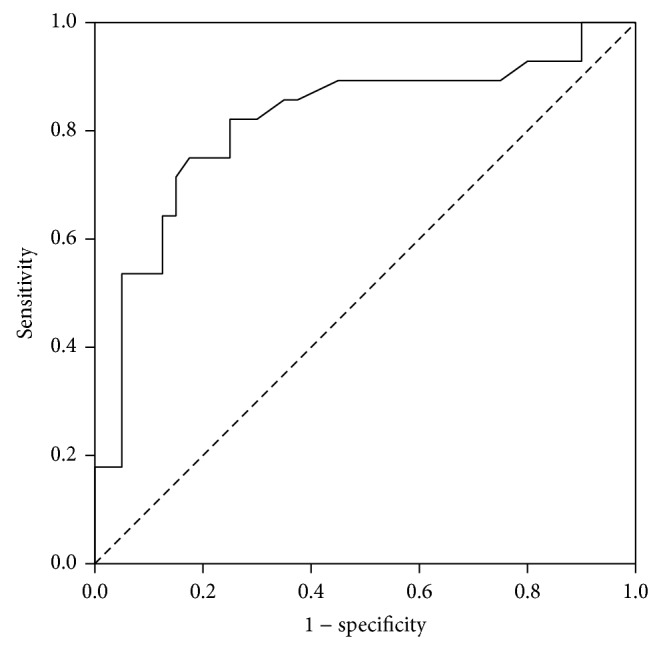
ROC curve for lactate.

**Figure 2:**
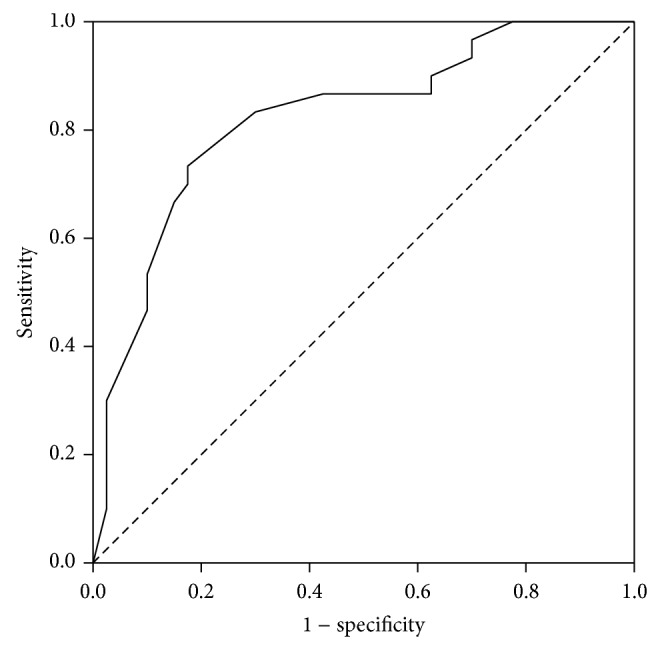
ROC curve for ISS.

The ROC curve for both lactate and ISS demonstrated good discriminating ability for predicting mortality with AUC for lactate of 0.82 (95% CI = 0.70–0.93, *P* < 0.001) and AUC for ISS of 0.82 (95% CI = 0.71–0.93, *P* < 0.001) (Figures 1 and 2). We determined a lactate level of 3.4 mmol/L as the optimal cut-off point. Lactate level of 3.4 mmol/l or more was 82% sensitive (95% CI = 63 to 94%) and 75% specific (95% CI = 59 to 87%) for prediction of in-hospital death, with a PPV of 70% (95% CI = 51 to 84%) and a NPV of 86% (95% CI = 70 to 95%). ISS of 30 or more was 73% sensitive (95% CI = 54 to 88%) and 83% specific (95% CI = 67 to 93%) for prediction of in-hospital death, with a PPV of 76% (95% CI = 56 to 90%) and a NPV of 81% (95% CI = 65 to 91%).

**Figure 3:**
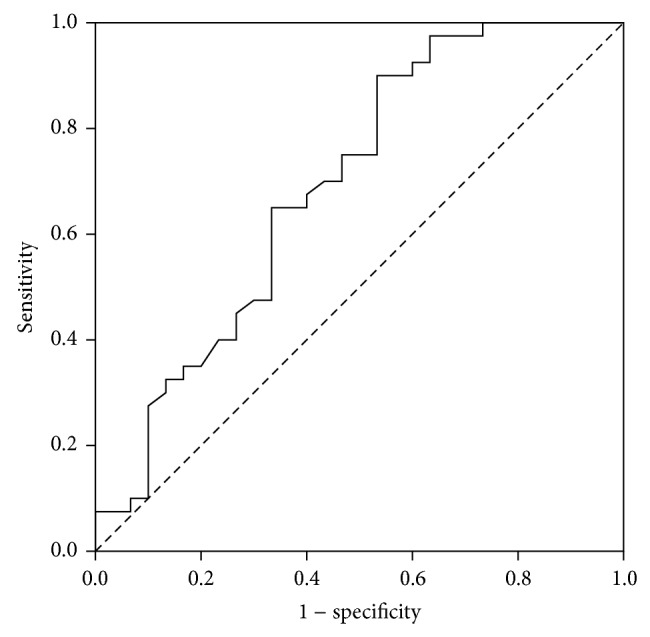
ROC curve for BD.

The ROC curve for BD demonstrated a poor discriminating ability for predicting mortality with AUC of 0.69 (95% CI = 0.56–0.82, *P* = 0.006) (Figure 3). We established a BD level of −9.25 as the optimal cut-off point. BD level of −9.25 or more was 47% sensitive (95% CI = 28 to 66%) and 90% specific (95% CI = 76 to 97%) for prediction of in-hospital death, with a PPV of 78% (95% CI = 52 to 93%) and a NPV of 69% (95% CI = 55 to 81%).

**Figure 4:**
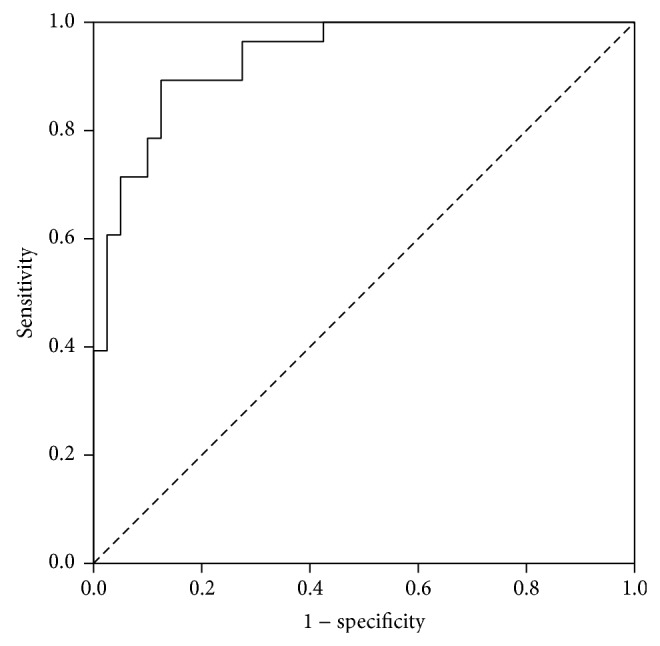
ROC curve for multivariate logistic regression model.

Results from multivariate logistic regression model analysis from our study show that blood lactate concentration on admission (*P* = 0.238) is not an independent predictor of mortality. However, ISS (*P* = 0.037), initial GCS (0.033), and age (0.002) from the same analysis did prove to be an independent prognostic factor. The logistic regression model fitted the data very well (likelihood-ratio test *P* < 0.001, Nagelkerke *R* ^2^ = 0.686) (Table 2). The model explained 68.6% of the variance in death and correctly classified 83.8% of cases. Increasing age, ISS, lactate, and pCO_2_ were associated with an increased likelihood of death. Odds ratios from our study indicated that lower (more negative) values of BD and lower values of initial GCS decrease the likelihood of a favourable outcome. The ROC curve demonstrates good discriminating ability for regression model with an AUC of 0.93 (95% CI = 0.88–0.99, *P* < 0.001) (Figure 4). Correlation between lactate and base deficit on admission (*r* = −0.743, *R* ^2^ = 0.552) was statistically significant.

## 4. Discussion

This study investigates independent predictors of in-hospital mortality in blunt trauma patients intubated in prehospital emergency care and prehospital monitoring limitations in these patients. Our results show that age, lactate levels, RTS, ISS, pCO_2_, initial GCS, and BD are associated with increased mortality. On the other hand, only age, ISS, and initial GCS from the multivariate analysis were found to be independent predictors of in-hospital mortality in the present study sample. Lactate levels were significantly higher in nonsurvivor group indicating more severe tissue hypoperfusion compared to the survivor group of patients. Inadequate organ perfusion after severe traumatic injury leads to oxygen debt and increase in serum lactate levels reflecting anaerobic metabolism. The duration of occult hypoperfusion and therefore continued elevation of serum lactate levels correlates with increased incidence of death, multiorgan failure, and respiratory complications after severe blunt trauma. On the other hand, early detection and rapid correction of lactic acidosis improve survival and decrease the incidence of multiorgan failure and respiratory complications [4].

Similar admission lactate levels and statistically significant difference in admission lactate levels between compared groups from the present study were also found in other studies [3, 9, 15]. Lactate was found as a modest predictor of outcome with AUC from 0.69 to 0.73 [6, 9, 12, 15]. Our data showed better accuracy of lactate as a predictor of mortality with AUC of 0.82. This fact could be due to some differences between study populations in the above-mentioned studies. Only severely injured blunt trauma patients with multiple injuries were enrolled in our study compared to study by Pal et al. [6] who also included patients with penetrating injuries. Jansen et al. [9] studied a population of patients with diverse medical conditions with trauma patients representing only a minor part (15%), whereas in the study by Tobias et al. [12] trauma patients were excluded. Nevertheless, results from multivariate logistic regression analysis from our study show that lactate level on admission is not an independent predictor of mortality. This finding is in accordance with other studies which show that lactate level is a poor independent predictor of mortality since admission lactate level above 2 mmol/L has a PPV of 4% [6] and with prehospital lactate cut-off point of 3.5 mmol/L the PPV increases to 41% for predicting death [9]. Our data showed higher PPV of 70% with lactate cut-off value of 3.4 mmol/L compared to previously mentioned studies. This finding could also be due to different study populations. Only the difference between prehospital lactate level and lactate level on arrival at the ED was reported as an independent predictor of in-hospital mortality [9]. Also, blood lactate concentration 12 hours after admission to the ICU is a good predictor of patient's survival [3].

Values of admission BD in the present study were significantly different between compared groups and indicate more severe hypovolemic shock in the nonsurvivor group. BD is associated with fluid requirements during resuscitation: more severe base deficit requires more fluid for resuscitation [8]. In previous studies, BD has been shown to be a valuable indicator of shock and the efficacy of resuscitation and is more reflective of the true volume deficit [16]. Admission BD also identifies patients who require early transfusions with more transfused blood products in patients with lowest BD and corresponds to increased rates of acute respiratory distress syndrome, multiorgan failure, and mortality [17]. It can also be used to identify patients with high risk of mortality who would require admission to the ICU [18]. Nevertheless, predictive ability of BD for mortality in our study is similarly poor as in other studies with AUCs of 0.65 and 0.70, respectively [5, 19]. Median values of BD in nonsurvivor group from the presented study were −7.3 with volume requirements in the first hour around 4000 mL according to Davis et al. [8]. Patients in our study group were resuscitated with approximately 1000 mL (median) volume in the prehospital phase. Also, the survivor group was resuscitated with volume replacement of median 1000 mL, but with requirements of around 3000 mL according to Davis et al. [8]. Decision making of Emergency Medical Service (EMS) system personnel depends on clinical findings and vital signs of the patient which are not reliable indicators of occult hypoperfusion [1, 2]. There were no significant differences in vital signs between compared groups in the present study. Furthermore, measurements of initial petCO_2_, which is a very reliable hemodynamic parameter used as noninvasive monitoring for evaluation of therapeutic efforts in prehospital environment in various clinical settings [20, 21], were in normal range in both groups. There was clear evidence of occult hypoperfusion (elevated lactate and lower values of BD) in both groups of patients in the presented study with no difference in vital signs and petCO_2_ values (in normal range) indicating that measurements in prehospital setting are insufficient to detect occult hypoperfusion [1, 7]. There is an existential need to be equipped with additional tools to recognize occult hypoperfusion in prehospital setting and to resuscitate trauma patients properly. It is possible to make fast and accurate measurements of lactate with hand-held analyzer [22]. In addition, serum lactate levels measured in the prehospital environment improved prediction of mortality and multiorgan failure and can be used for identification of patients who require more aggressive early resuscitation [13]. It was also shown that prehospital blood lactate level measurements provide prognostic information about in-hospital mortality superior to that provided by the patient's prehospital vital signs and could be used to guide prehospital resuscitation [9]. Similarly, lactate-guided therapy in ICU significantly reduces hospital mortality [10]. Furthermore, the time needed to normalize serum lactate levels is an important prognostic factor for survival [7]. It was also shown that early lactate clearance in the first two hours after admission is an important and independent prognostic variable [23].

Findings from our study also show that ISS and initial GCS were independent predictors of outcome. Survivors and nonsurvivors also differed significantly in their physiological statuses assessed by the RTS. This observation indicates that nonsurvivors were more severely injured and neurologically compromised compared to survivors and is in accordance with other studies which showed that an ISS > 20 has a greater discriminating ability than lactate [4] and that prehospital GCS score is an independent predictor of in-hospital mortality [7]. Furthermore, there is a clear association between lactate concentrations on admission [1] and on the third day of ICU treatment [24] with ISS. Values of ISS and RTS in survivors and nonsurvivors in our series were similar as in other studies [3, 24]. It was also shown that trauma patients with higher ISS also had prolonged occult hypoperfusion (more than 24 hours) and therefore higher infection rate, predominating lung infections [25]. In addition, anatomic injury scoring systems grading the severity of trauma patients have predictive value for complications such as acute respiratory syndrome and multiple organ failure [24].

Age is confirmed as an independent predictor of outcome. Elderly patients (age above 65) have a nearly twofold increase in mortality risk and have significantly longer hospital length of stay [26]. Mortality in elderly could be predicted by lactate [27], ISS, and medical complications [28], whereas chest complications and dysrhythmias are most frequent [29]. Preexisting medical conditions (PMC) in the elderly are associated with increased mortality after low to moderate severity injuries, but not after more severe injuries [30]. This is explained by diminishing respiratory and cardiovascular reserve, PMC, and medications [31]. Elderly patients with minor injuries and PMC also have increased risk of late death from medical complications that are not directly related to their original injury [32]. Our study showed and supported the above-mentioned studies that age is an independent factor of mortality and should be considered in contemporary scoring systems and treatment algorithms for elderly patients, where early invasive hemodynamic monitoring could identify occult shock and prevent multiorgan failure and therefore improve survival [33].

Our study showed that patients who did not survive tend to be more hypercapnic compared to survivors who were normocapnic. Numerous studies have described the protective effects of hypercapnia in hemorrhagic shock [34]. Permissive hypoventilation leads to greater venous return, higher cardiac output, and systolic blood pressure and to lower lactic acid and serum creatinine levels suggesting better perfusion of vital organs compared to positive pressure ventilation in swine model of hemorrhagic shock due to penetrating injury [35]. It may also actually help resolve cerebral hypoxia induced by hemorrhagic shock [36].

### 4.1. Limitations

Our study is limited by the use of convenience sample of subjects who were severely injured due to blunt trauma and therefore intubated already in the prehospital emergency care. This group may be more severely injured compared to the general population of trauma patients transported by an ambulance. The association between lactate and other predictors among those who were not intubated in the prehospital emergency care was not assessed.

Secondly, this is a nonrandomized retrospective study. The nature of the study by itself could include some biases. In the present study analysis of the data collected from January 2000 to December 2012 was done. Progress over the years in in-hospital care of severely injured patients may affect the rate of in-hospital mortality. However, ambulance treatment protocol remained mostly the same throughout the study period.

Thirdly, the sample size is small.

## 5. Conclusions

The presented data from our study sample show that admission lactate, BD, pCO_2_, age, initial GCS, ISS, and RTS were associated with in-hospital mortality. Lactate was found to be a good predictor of in-hospital mortality, whereas BD provides poor discrimination of survival. Only age, ISS, and initial GCS were found as independent predictors of in-hospital mortality in our study sample. There was clear evidence of occult hypoperfusion (elevated lactate and lower values of BD) in both groups of patients with no difference in prehospital vital signs and petCO_2_ values (in normal range) indicating that measurements in prehospital setting are insufficient to detect occult hypoperfusion which was more profound in the nonsurvivor group. There is a need to be equipped with additional tools to recognize occult hypoperfusion in the prehospital setting and to resuscitate trauma patients properly. Larger prospective studies are necessary to validate these findings.

## Figures and Tables

**Table 1:** Patients' characteristics.

Variables	Survivors (*n* = 40)	Nonsurvivors (*n* = 30)	*P* value
Age (years)	29 (22–51)	50 (36–72)	0.006^1^
Male sex, *n* (%)	33 (83%)	21 (70%)	0.258^2^
petCO_2_ (mmHg)	38 (34–44)	38 (33–45)	0.926^1^
HR (min^−1^)	100 (92–110)	100 (90–106)	0.575^1^
MAP (mmHg)	90 (78–100)	86 (73–103)	0.821^1^
RR (min^−1^)	12 (10–20)	12 (9–16)	0.208^1^
SaO_2_ (%)	89 (79–93)	86 (80–95)	0.770^1^
Initial GCS	6 (5–11)	4 (3–7)	<0.001^1^
RTS	6.0 (4.7–6.9)	4.4 (4.1–5.7)	0.001^1^
ISS	24 (17–29)	36 (29–42)	<0.001^1^
Number of patients with head AIS ≥3	28 (70%)	20 (67%)	0.779^2^
Lactate (mmol/L)	2.6 (2.0–3.5)	5.3 (3.7–7.8)	<0.001^1^
BD (mmol/L)	−4.3 (−7.2–−1.7)	−7.1 (−18.1–−3.3)	0.006^1^
pCO_2_ (kPa)	5.4 (5.1–6.1)	6.1 (5.1–7.7)	0.026^1^
pO_2_ (kPa)	22.3 (14.3–36.1)	16.9 (9.4–34.2)	0.068^1^
Alcohol (g/L)	0.6 (0.0–2.1)	0.0 (0.0–0.7)	0.152^1^
Intervention duration (min)	47 (39–53)	49 (41–58)	0.776^1^
Drugs and fluids			
Etomidate (mg)	20 (20-20)	20 (20-20)	0.578^1^
Midazolam (mg)	5.0 (5.0–7.1)	5.0 (5.0-5.0)	0.241^1^
Fentanyl (*μ*g)	0.100 (0.100–0.163)	0.100 (0.100–0.138)	0.851^1^
Succinylcholine (mg)	100 (80–100)	100 (100-100)	0.081^1^
Vecuronium (mg)	8 (4–8)	4 (4–8)	0.016^1^
Piritramide (mg)	15.0 (12.5–15.0)	15.0 (11.3–15.0)	1.000^1^
6% HES (mL)	500 (500–1000)	500 (500–1000)	0.593^1^
0.9% NaCl	500 (500–1000)	500 (500–1000)	0.484^1^

Values are presented as median (IQR). IQR: interquartile range; petCO_2_: partial pressure of end tidal carbon dioxide; HR: heart rate; MAP: mean arterial pressure; RR: respiratory rate; SaO_2_: arterial oxygen saturation; GCS: Glasgow Coma Scale; RTS: Revised Trauma Score; ISS: Injury Severity Score; AIS: Abbreviated Injury Scale; BD: base deficit; pCO_2_: partial pressure of carbon dioxide; pO_2_: partial pressure of oxygen; HES: hydroxyethyl starch; NaCl: saline.

^1^Mann-Whitney *U* test, ^2^Fisher exact test.

**Table 2:** Results of multivariate logistic regression model of predicting in-hospital death^*^.

Variables	OR (95% CI)	*P* value
Age (years)	1.10 (1.02–1.12)	0.002
Initial GCS	0.71 (0.51–0.97)	0.033
ISS	1.11 (1.01–1.22)	0.037
Lactate (mmol/L)	1.34 (0.83–2.16)	0.238
BD (mmol/L)	0.95 (0.80–1.14)	0.587
pCO_2_ (kPa)	1.50 (0.90–2.50)	0.123

^*^Chi-square value = 48,406, df = 6, *P* < 0.001, and Nagelkerke *R* ^2^ = 0.686.

OR: odds ratio; CI: confidence interval; ISS: Injury Severity Score; BD: base deficit; pCO_2_: partial pressure of carbon dioxide; GCS: Glasgow Coma Scale.
